# Anatomical Comparative Study of the External Nasal Nerve in Caucasian and Asian: Application for Minimizing Nerve Damage in Rhinoplasty

**DOI:** 10.1007/s00266-021-02556-1

**Published:** 2021-09-08

**Authors:** Yue Chen, Catherine B. Carr

**Affiliations:** Centre for Anatomy and Human Identification, School of Science and Engineering, University of Dundee Nethergate, Dundee, DD1 4HN Scotland UK

**Keywords:** External nasal nerve, Anatomy, Rhinoplasty

## Abstract

**Background:**

The numbness of the nasal tip is the main symptom of the external nasal nerve injury, especially after rhinoplasty. This postoperative syndrome can reduce the patient’s satisfaction with the operation. Having a better understanding of the anatomical structure and intraoperative protection can effectively avoid nerve injury. At present, the anatomical research on this nerve is all from Asia. This study aims to fill the gap in the anatomical study of this nerve in Caucasians and provides comparative results with Asians.

**Material and Methods:**

A total of 20 Caucasian cadavers were embalmed using the Thiel method. On dissection, after complete exposure of the external nasal nerves, the distance between the exit point of the external nasal nerve and the nasal midline was measured, and the morphology of the nerves was compared with the Asian data. The nerves were classified into types based on their branching pattern.

**Results:**

The nerve plane was the same as the Asian record. The distance ranged from 5.08 to 11.94 mm (mean, 8.31 ± 1.85 mm). This distance has statistical significant difference compared with the Asian population (*P* < 0.01). The average distance is larger, and the distribution range of the exit point is wider. On classification, 35 of 40 cases had the same type results as those previously reported, with the primary types I, II and III. Five new varieties were found which are classified as subtypes of the primary types and a new type IV. Furthermore, the bifurcation position in two-thirds of the type II cases and variations is proximal to that seen in the Asian population.

**Conclusions:**

The anatomical structure of the external nasal nerve in Caucasians and Asians has obvious differences. This nerve in Caucasians is more likely to be damaged during rhinoplasty than Asians. Except the primary types, the classification of the external nasal nerve also includes subtypes and type IV.

**Level of Evidence III:**

This journal requires that authors assign a level of evidence to each article. For a full description of these evidence-based medicine ratings, please refer to the Table of Contents or the online Instructions to Authors www.springer.com/00266.

## Background and Aim

The nose plays a central role in determining overall facial esthetics. In pursuit of facial esthetics, some people choose to meet their requirements through rhinoplasty [1]. In Caucasians, reduction rhinoplasty with volume, dorsal hump and lower lateral cartilage is more popular than that in Asians [2]. Furthermore, female Caucasian patients frequently desire a more slender nasal tip and that prompts surgical consultation about the rhinoplasty of shaping of the nasal tip [1].

After rhinoplasty, many patients report postoperative complications. Nasal tip numbness is the main postoperative complication of external nasal nerve damage. A study by Thompson [3] showed that more than half of the patients had nasal tip numbness after rhinoplasty, especially those with nose tip reduction. The twin-center study from Jaberoo et al. [4] shows that 26.2% of patients experience postoperative nasal tip numbness, with duration ranging from two weeks to more than one year. This symptom is mainly caused by injury to the external nasal nerve which supplies sensation to the distal aspect of the nasal dorsum and tip of the nose, as well as the skin of the nasal ala [5]. Furthermore, a series of rare symptoms, known as Charlin’s syndrome, have also been reported in a patient who experienced external nasal nerve damage after a routine septorhinoplasty [6]. The symptoms include upper facial pain, headache and ipsilateral rhinorrhea [7]. Besides, neuralgic pain and hyperesthesia during nerve recovery also occur which are caused by stump neuroma formation after the nerve is severed [8]. These postoperative complications can greatly reduce the patient’s satisfaction with the operation, even if the operation itself is completed to a high standard.

The treatment of external nasal nerve injury is currently based on symptomatic treatment, supplemented by etiological treatment. In most cases, the recovery of sensation depends on the regrowth of the severed nerve or collateral sprouting from the adjacent nerve [9]. Etiological treatment is generally applied to the discomfort caused by nerve compression. For instance, if the pressure is caused by the placement of the implant, removing the implant can improve the symptoms of nerve compression [10]. However, there is no effective treatment for when the nerve is severed. Symptomatic treatment is mainly used to alleviate symptoms such as Charlin’s syndrome. Treatment includes nasociliary ganglion block with local anesthetic and steroids [6]. Furthermore, the definitive and permanent therapy is chemical ablation or surgical transection of the nasociliary or anterior ethmoidal nerves [11]. But this therapy remains a matter of debate. Besides, the individual prognosis varies greatly. For instance, Thompson [3] reported that nearly one-third of the 75 patients studied after rhinoplasty experienced only numbness of the nasal tip, and more than half of them recovered within three months. However, Kavyani et al. [6] reported a case of Charlin’s syndrome whose symptoms persisted for four years and gradually intensified. Thus, there is currently no clear and effective treatment for external nasal nerve injury. At the same time, there are huge individual differences in the prognosis.

There is currently no ideal treatment for postoperative complications. Moreover, if the operation area has scar hyperplasia, under blind vision, capillary bleeding blurred operation area and so on, which makes the operation more difficult, the possibility of damaging the external nasal nerve will be extremely high in clinical works. So it is necessary to have a better understanding of this nerve to avoid nerve injury. However, so far, there are few anatomical records for this nerve, and they are all from Asian populations. Therefore, this study of Caucasians can complement the anatomical study of the external nasal nerve. The aim of this study is to describe the course of the external nasal nerve in a Caucasian population and compare this with previously published data on the Asian population. It also uses measurement data to propose specific methods for avoiding nerve damage during surgery.

## Materials and Methods

A total of 20 cadavers were acquired from The Centre for Anatomy and Human Identification (CAHID), University of Dundee. The cadavers were all Caucasian and were embalmed using the Thiel method. The residence of all individuals at their demise was Scotland. Each cadaver dissected in this study was numbered and photographed to avoid confusion. All cadaveric research was conducted in full compliance with relevant anatomical legislation, with all donors having given their consent in accordance with the Anatomy Act (1984) and the Human Tissue (Scotland) Act (2006). The specific information of the individuals is provided in Table 1. The sample is made up of 9 female and 11 male cadavers. The age range of the individuals was 62 to 97 (average age: 80). The superficial facial areas of these 20 individuals were at least partially dissected at an earlier date by students in CAHID. However, there was no deficiency and damage to the external nasal nerves. There was no obvious deformity of the nose shape. The statistical data analysis is carried out by SPSS 26.0.

**Table 1:** Specific information for the 20 cadavers.

Age	Sex	Cause of death
62	M	Metastatic disease, prostate adenocarcinoma, right leg deep Vein Thrombosis
63	M	Myocardial infarction, type 1 diabetes, psoriatic arthritis, HypertensiveDisease
72	F	Dementia
73	M	Chest infection, pulmonary fibrosis, COPD
73	F	Metastatic breast cancer, hypertension, atrial fibrillation, Hypothyroidism
74	M	Squamous cell carcinoma of the lung
75	M	Squamous cell carcinoma of Tongue, COPD, ischemic heart Disease
77	F	COPD, heart/lung failure, type II DIABETES
79	M	Right upper lobe lung carcinoma with adrenal, liver and brain metastases
79	M	End stage dementia, diabetes, hypertension, IGA
82	F	Myocardial infarction, hypertensive disease, AF, Type II Diabetes, Bronchiectasis
83	M	Community-acquired pneumonia
84	M	Alzheimer’s disease
84	F	Heart failure, pneumonia, COPD, neck of femur fracture
86	F	Parkinson’s disease
86	M	Vascular dementia, type II diabetes
87	F	Community-acquired pneumonia, congestive cardiac failure
90	F	Atrial fibrillation, hypertension, cardio-respiratory arrest
94	M	Bronchial pneumonia, myasthenia gravis, fractured neck of Femur
97	F	left ventricular failure, myocardial infarction, coronary artery Disease, Vascular Dementia
Average: 80	M/F: 11/9	

**Fig. 1:**
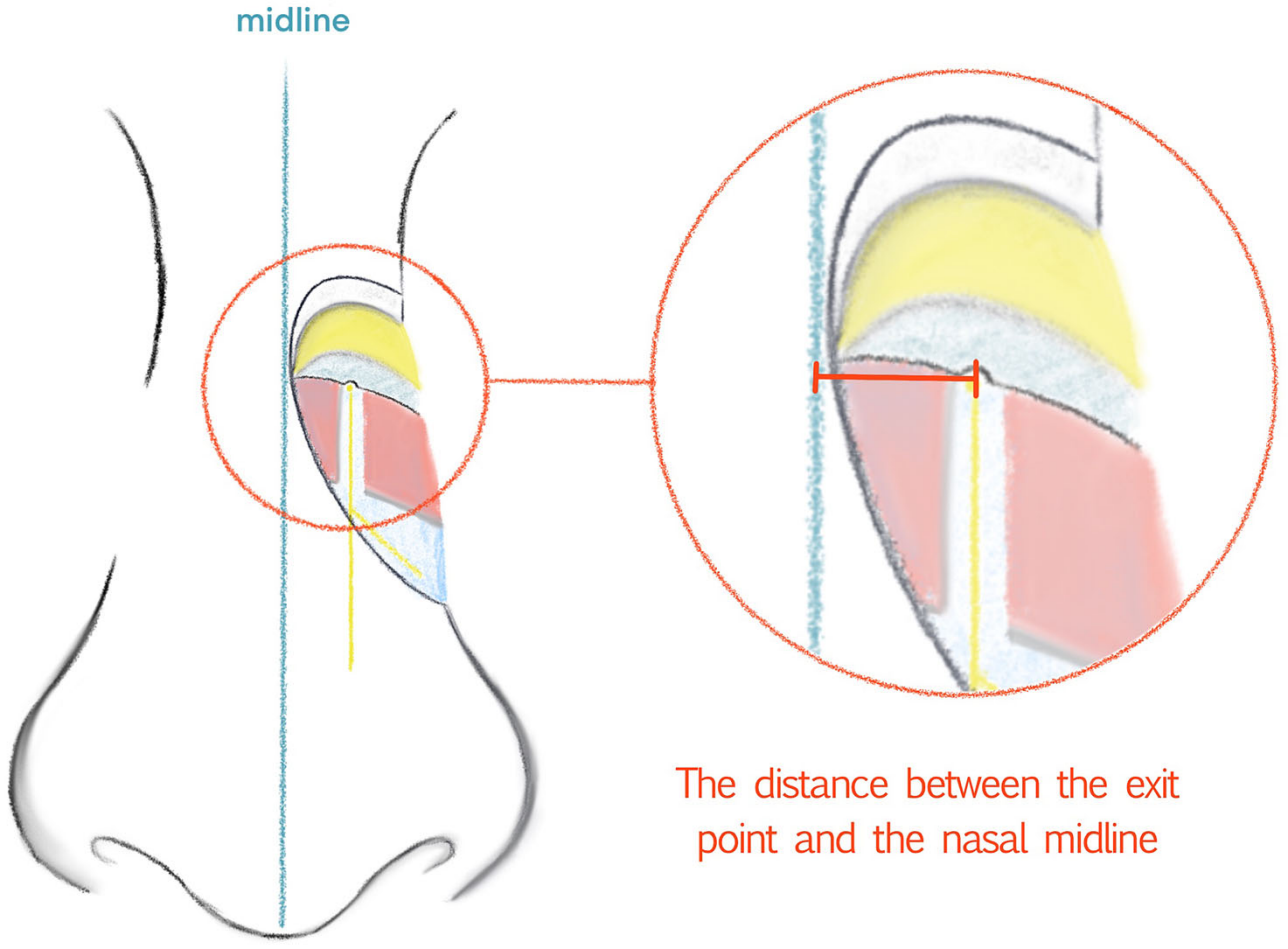
The distance to be measured is indicated by the thick orange line

Before dissection, the nasal midline was first marked. From the midline, the dorsal skin, subcutaneous fat tissue, transverse nasalis and deep fatty layer were dissected gradually using standard clinical surgical instruments. After separating the transverse nasalis, the exit point and course of the nerve could be clearly seen. Between the nasal bone and the upper lateral cartilage, the exit point was identified. After complete exposure of the external nasal nerves on both sides, a dial caliper with an accuracy of 0.02 mm was used to measure the distance between the exit point of the external nasal nerve and the nasal midline to two decimal places. The position where the measurement was taken is shown in Fig. 1. At the same time, the course of nerve was classified according to the classification method proposed by Han et al. [12], classifying types by nerve branching pattern. In addition, the variation in the nerves was also recorded, including the number of branches and the bifurcation positions. The results were compared with previous anatomical studies from Korea [12] and China [13].

## Results

**Fig. 2:**
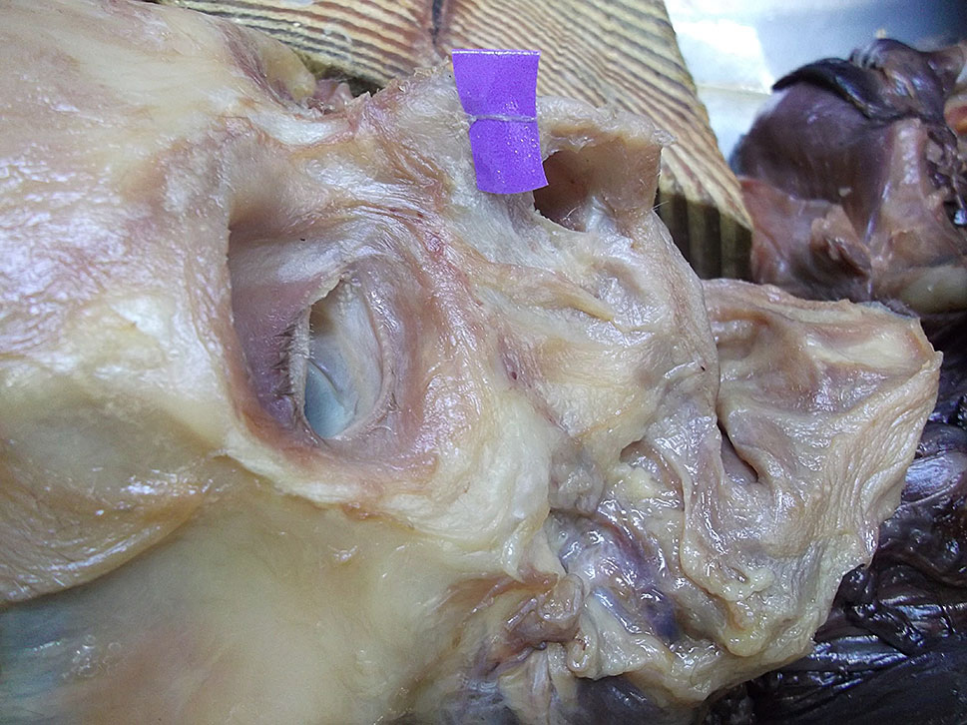
The shortest distance is 5.08 mm (Right side)

**Fig. 3:**
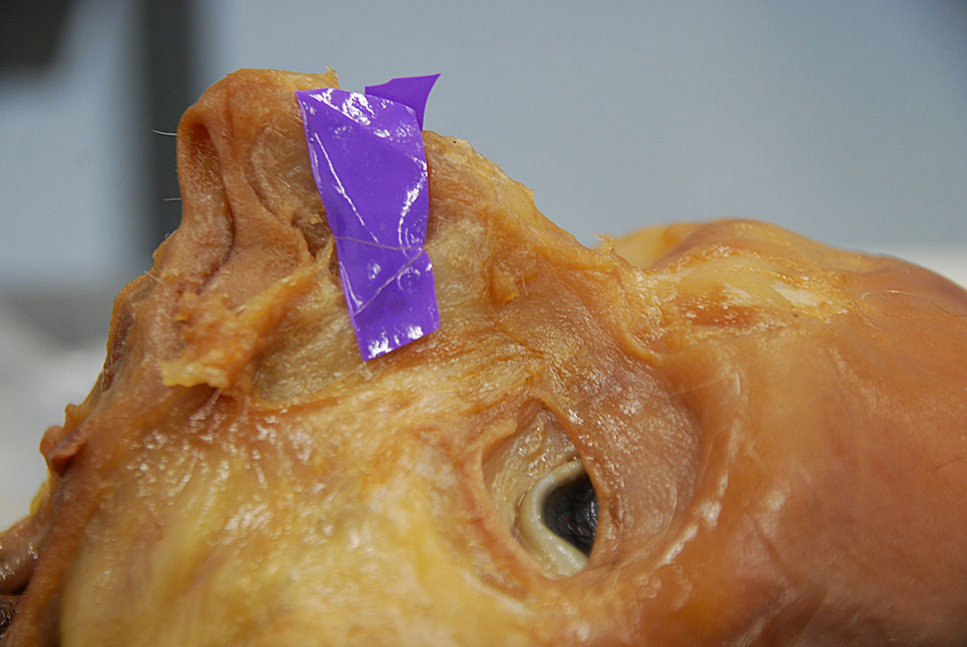
The longest distance is 11.94 mm (Left side)

**Fig. 4:**
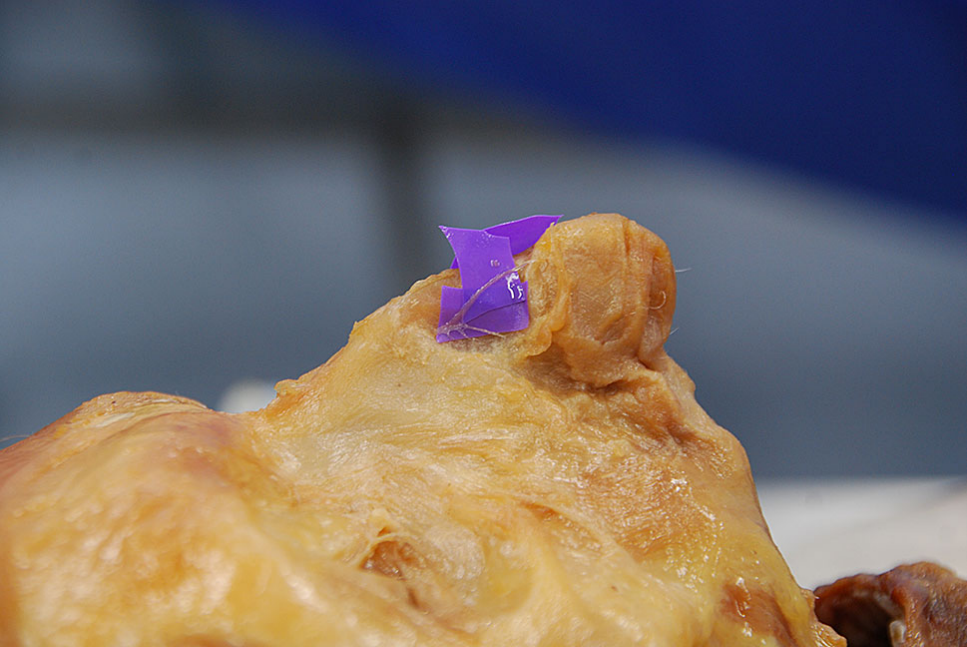
Uncertain type 1. (Right side)

**Fig. 5:**
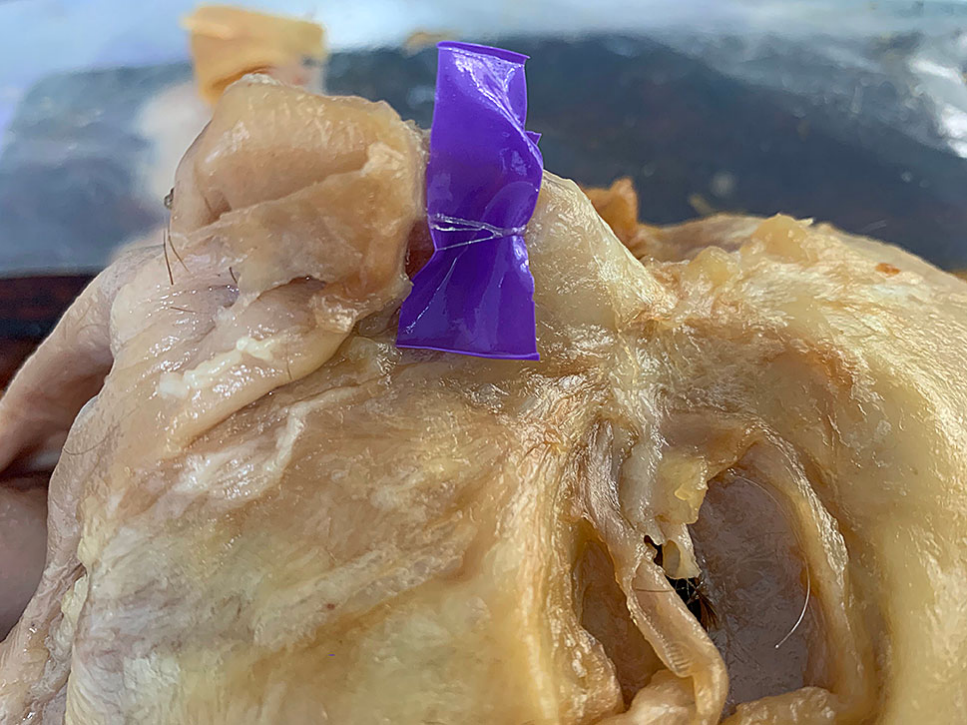
Uncertain type 2. (Left side)

**Fig. 6:**
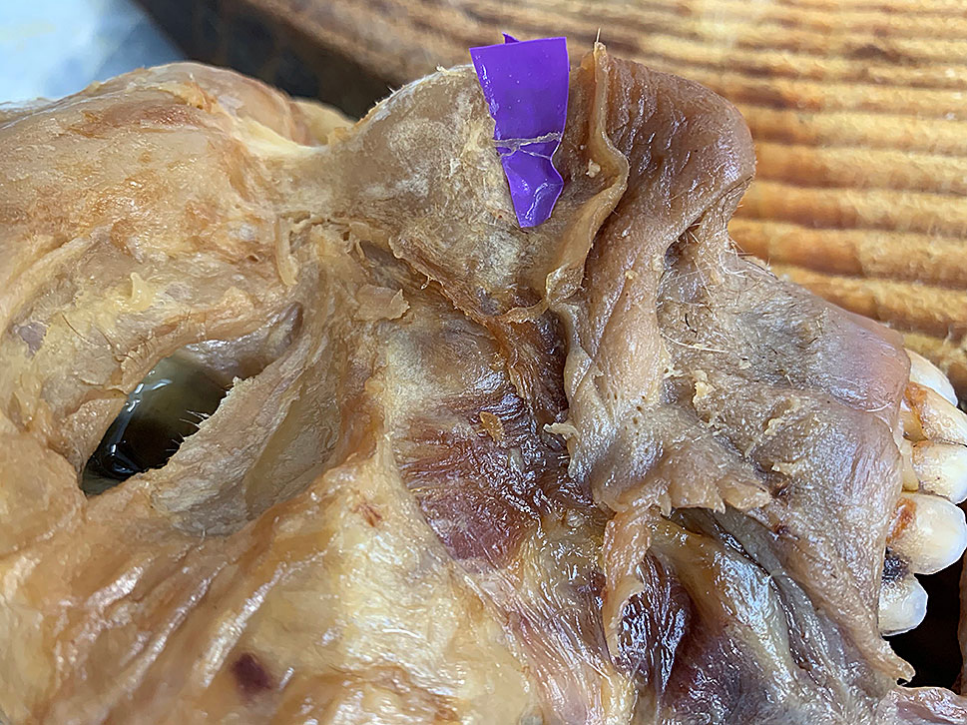
Uncertain type 3. (Right side)

**Fig. 7:**
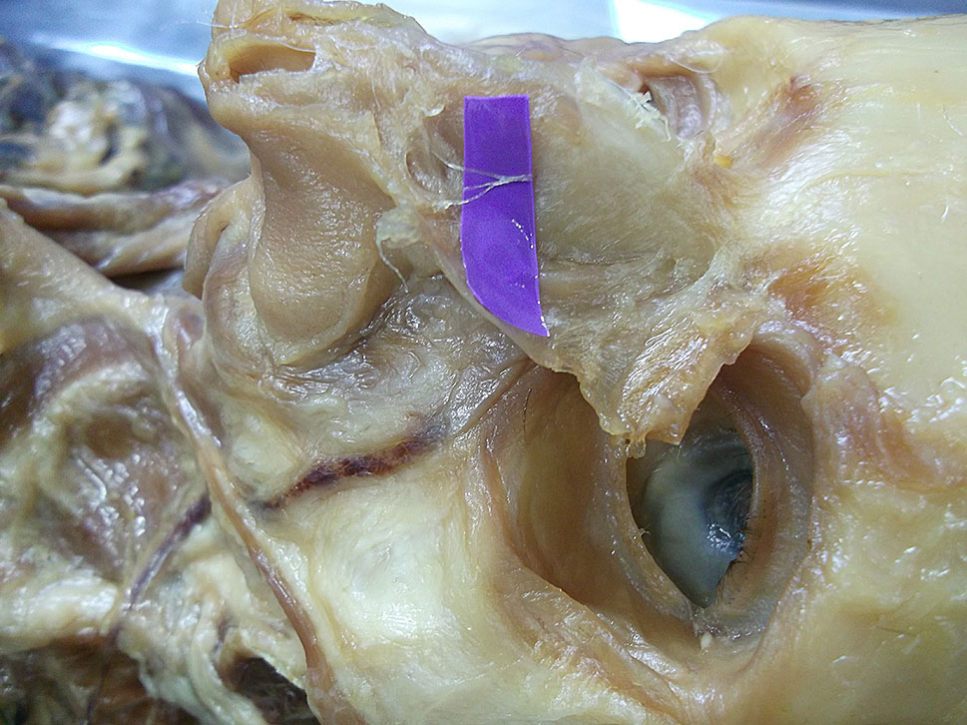
Uncertain type 4. (Left side). The morphology of the uncertain type 5 is highly similar to the uncertain type 4 and is also on the left

**Fig. 8:**
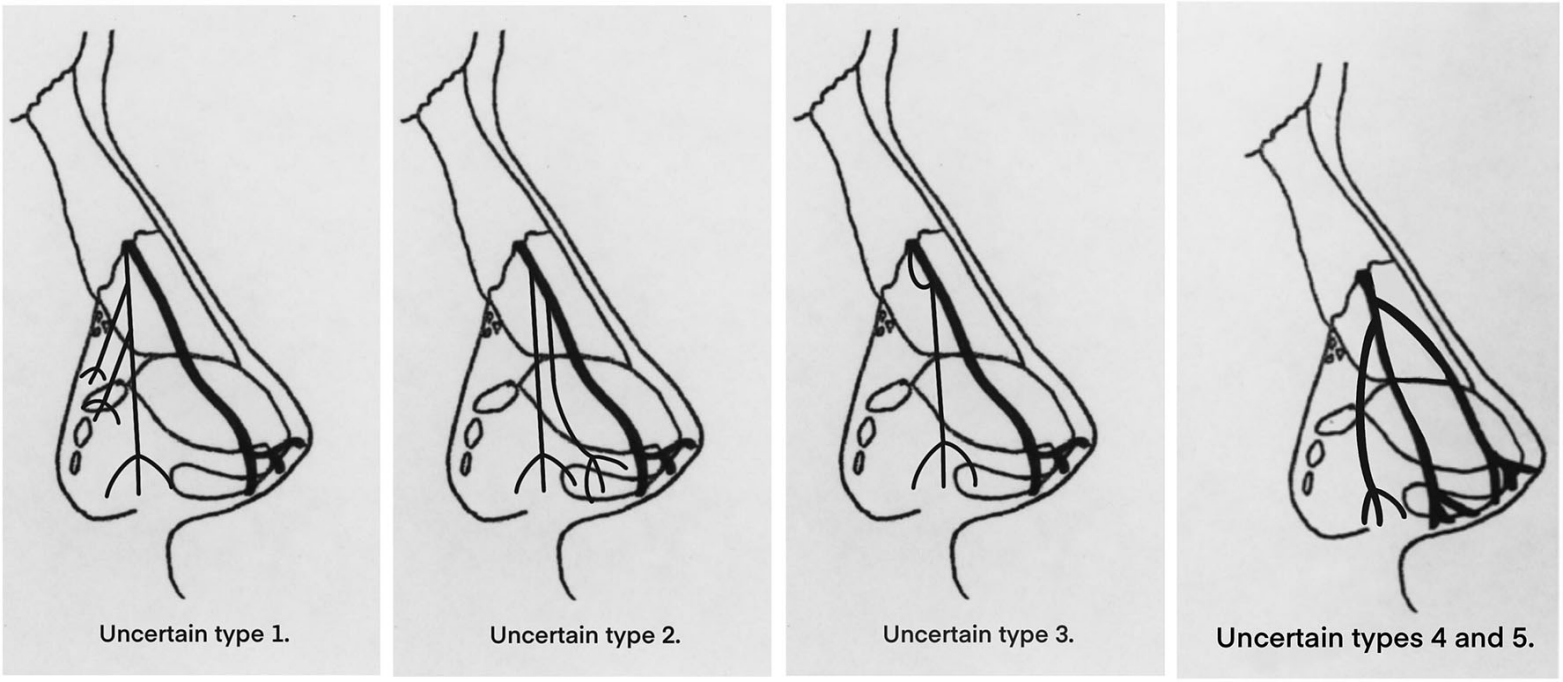
Drawing illustrating the nerve branching of uncertain types 1–5. Uncertain types 1–3 have different bifurcation patterns compared to the types defined by Han et al. [12] uncertain types 4–5 have the same bifurcation pattern.

The external nasal nerves from the 20 individuals were found on both sides, and no absence of nerves occurred. After emerging from the exit point, all the nerves were found beneath the transverse nasalis, on the perichondrium, and passing within the deep fatty layer. In some individuals, a small notch was found on the nasal bone beside the exit point of the nerve. The main branch coursed downward beside the nasal midline and ended at the apex of the nostril. The distance to the midline ranged from 5.08 to 11.94 mm (mean, 8.31 ± 1.85 mm). The shortest and the longest distances are displayed in Figs. 2, 3. Among the three basic types, type I had the most cases (25), accounting for 62.5% of the total 40 cases; type II followed with 6 cases, accounting for 15% and Type III was the rarest, with 4 cases, accounting for 10.0%. Additionally, there were 5 cases of unknown type, two of which had the same branching pattern, accounting for 12.5%. These variations are displayed in Figs. 4, 5, 6, 7. According to the morphology of these five uncertain types, a schematic diagram illustrating the nerve branching is drawn in Fig. 8. The measured distances and the external nasal nerve types from all individuals are provided in Table 2. Nerves that do not fit any of the three classifications are listed as ‘uncertain’ in Table 2.

**Table 2:** The distance from the point of exit to the nasal midline at the osseocartilaginous junction and the classification of branching pattern type for each nerve.

	Basic information		Point of Exit			
	Age (year)	Sex	Left (mm)	Type	Right (mm)	Type
	62	M	9.02	*Uncertain 5*	5.32	I
	63	M	10.78	I	9.22	I
	72	F	5.24	I	6.38	I
	73	M	*11.94*	III	8.9	*Uncertain 1*
	73	F	11.82	I	11.18	I
	74	M	10.64	I	9.04	I
	75	M	10.1	I	10.1	I
	77	F	6.16	II	5.82	I
	79	M	6.52	I	7.08	I
	79	M	9.32	III	7.9	I
	82	F	9.44	III	9.74	II
	83	M	8.16	*Uncertain 4*	7.84	III
	84	M	7.72	I	8.8	*Uncertain 3*
	84	F	7.74	I	*5.08*	I
	86	F	7.02	*Uncertain 2*	7.3	I
	86	M	9.1	I	6.62	I
	87	F	7.82	I	7.2	I
	90	F	11.42	I	7.06	II
	94	M	9.96	II	6.8	I
	97	F	8.88	II	6.14	II
Average	80		8.94		7.68	
				8.31		
Mean ± SD			8.94 ± 1.85 ( *n* = 20 )		7.68 ± 1.62 (*n* = 20 )	
				8.31 ± 1.85 ( *n* = 40 )		

The longest value, the shortest value and the uncertain types are marked in italic.

## Discussion

The external nasal nerve originates from the trigeminal nerve (CN V). It belongs to the anterior ethmoidal nerve, which is a terminal division of the nasociliary nerve, a branch of the ophthalmic division (CN V1) of the trigeminal nerve [14]. In the literature, Han et al. [12] first proposed that the external nasal nerve could be classified into three main types. The types were classified according to their branching patterns: ‘type I, only one nerve without any branch; type II, one nerve proximally and then splitting into two main branches at the intercartilaginous junction; and type III, two main branches from the point of exit.’ [12] A further anatomical study from Tian et al. [13] agrees with Han et al.’s classification method. In their study, they found only type I and type II, but no type III. Table 3 shows the number and percentage of the total sample size of the three basic types in this study compared with the data values from Asia. The measurement data of distance and the comparative value in this study are presented in Table 4. The results of the independent sample T test of the exit point distance are shown in Table 5. The data of this study were compared with Korea and China, respectively. And the P values of both groups are less than 0.01. This distance has statistical significant difference between Caucasian and Asian populations.

**Table 3:** The number of types of external nasal nerves and their percentage in the sample size of this study and the comparative value from Asian [12, 13]

	Caucasian		Asian			
	In this study		Korean		Chinese	
	Number	Percentage (*n* = 40)	Number	Percentage (*n* = 20)	Number	Percentage (*n* = 38)
Type I	25	62.5%	10	50.0%	24	63.2%
Type II	6	15.0%	6	30.0%	14	36.8%
Type III	4	10.0%	4	20.0%	–	–
Uncertain	5	12.5%	–	–	–	–

**Table 4:** The measurement data of this study is compared with the data from Koreans and the data from Chinese [12, 13]

	Sample size	Embalming method	Shortest (mm)	Longest (mm)	Mean ± SD (mm)
This study	20 cadavers (40 nerves)	Thiel method	5.08	11.94	8.31 ± 1.85
Korean	10 cadavers (20 nerves)	Fresh body	6.50	8.50	7.30 ± 0.60
Chinese	20 cadavers (38 nerves)	10% formalin	5.40	6.80	5.90 ± 0.47

**Table 5:** The results of the independent sample T test of the distance between the external nasal nerve exit point and the nasal midline

Mean ± SD				T	Sig	P
Caucasian		Asian				
This study	8.31 ± 1.85	Korean	7.30 ± 0.60	3.138	0.003	< 0.01
		Chinese	5.90 ± 0.47	7.973	0.000	< 0.01

**Fig. 9:**
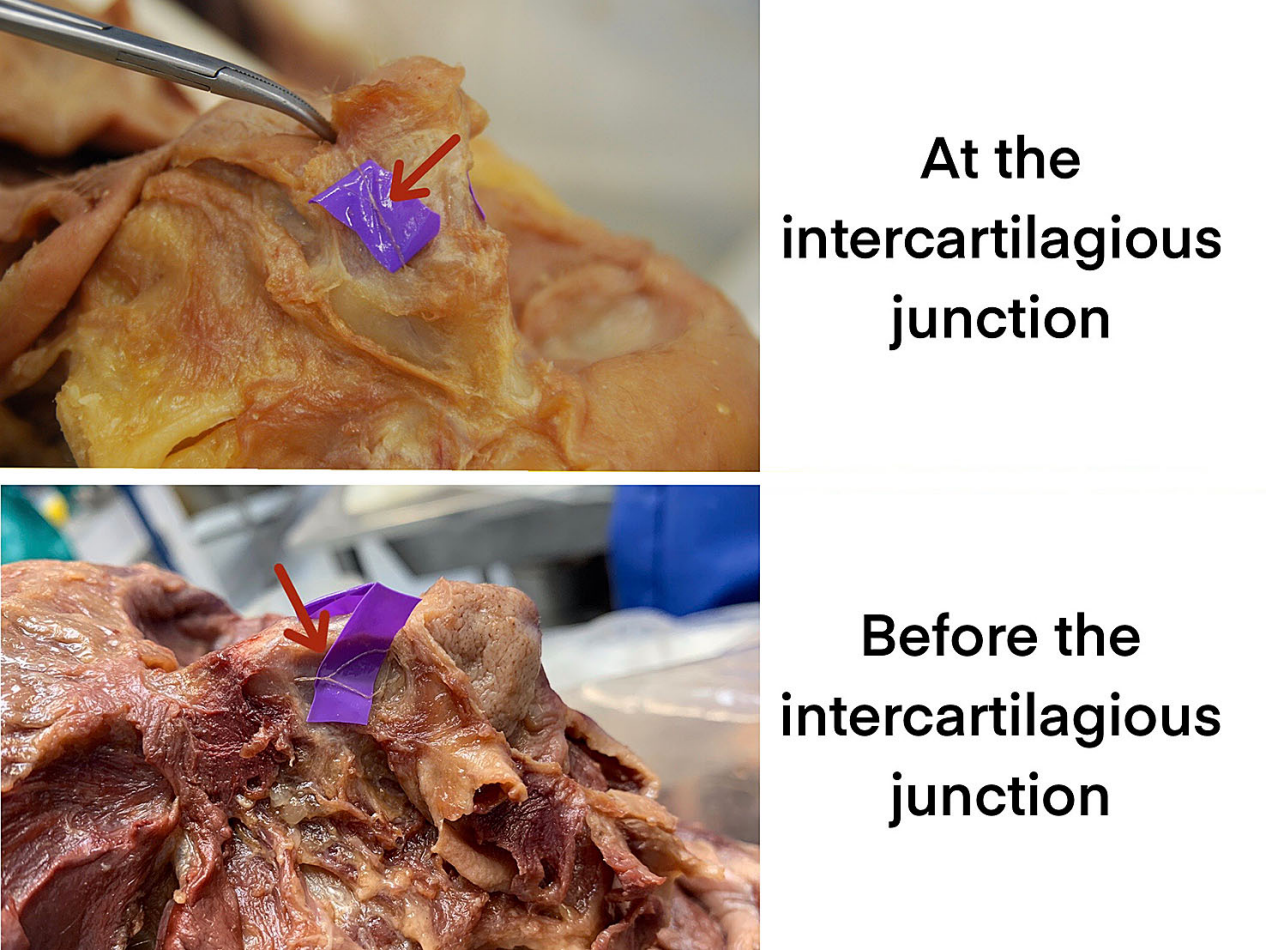
These two kinds of type II are bifurcated at or before the intercartilagious junction between the upper lateral cartilage and the greater alar cartilage

Differences in bifurcation positions were found in nerves classified as Type II, and new varieties were also found in addition to the three primary classification types. These new varieties are classified as Type IV. The bifurcation position from Han *et al.*’s research suggested that in type II, the bifurcation is at the intercartilaginous junction [12]. In this study, the same branching patterns of Types I, II and III were all found. The proportion is also similar with the previous anatomical studies. However, in a total of 6 cases of type II nerves, only 2 cases bifurcated at the intercartilaginous junction. Four bifurcated at a position between the exit point and the intercartilaginous junction. In other words, the bifurcation point of external nasal nerve type II in this study was most commonly located at a more proximal position than the record from the Asian population. These two kinds of Type II are provided in Fig. 9.

The five uncertain cases could not be classified by Han *et al*.’s method. Uncertain type 1 is considered as a subtype of type III. In this type, the nerve bifurcates at the exit point but the branches are a thick main branch (medial branch) and a thin secondary branch (lateral branch). Since the first branch of this variation is also the perforation point nerve, the difference is that the secondary (lateral) branch is thinner than the main branch (medial), and the lateral branch has more branches while the main branch does not. Therefore, this classification is a subtype of type III. Uncertain type 2 is considered as a subtype of type I. The course of the main branch of the uncertain type 2 is similar to Type I, which is distributed from the proximal exit point to the tip of the nose. However, two tiny branches were separated from the main branch before the intercartilaginous junction and were distributed in the greater alar cartilage and fibro-fatty tissue along the lateral ala. Therefore, the classification of uncertain type 2 can be considered as a subtype of type I. Uncertain type 3 is considered as a subtype of Type II. It has a recurrent branch which was considered as one of the two lateral internal nasal nerve branches separated from the anterior ethmoid nerve. Both lateral internal nasal branches emerged from the exit point then they joined together as one external nasal nerve. Additionally, the secondary nerve bifurcation position is also proximal to the intercartilaginous junction. Therefore, this uncertain type is more like a subtype of type II, but the bifurcation is located closer to the proximal nerve outlet. Uncertain types 4 and 5 are two similar types that cannot be classified by branch position and number as basic types. Both types gave off three equally thick branches just before the intercartilaginous junction. The medial branch coursed straight downward alongside the nasal midline and ended at the apex of the nostril. The middle branch went in the lateral of the nasal alar, passed the intercartilaginous junction and was distributed at the greater alar cartilage. The lateral branch went to the lateral side of the nasal alar, passed the lesser alar cartilage and was distributed in the fibro-fatty tissue. Therefore, the lateral branch may contribute to the sensory innervation of the alar part of the nose with the infra-orbital distal branches. Since these two cases cannot be classified into any of the three basic types, according to the position of bifurcation and branch number, they have been classified into a new type, Type IV. These five variations are presented in Table 6.

**Table 6:** Classification of the five variations.

1.	2.	3.	4.	5.
				
Subtype of type III	Subtype of type I	Subtype of type II	type IV	

On the bifurcation point, except for type I and type III, all type II (6) and all variations (5) have branches after the nerve exit point. And in these 11 cases, only 2 cases (18.2%) in type II bifurcated at the intercartilaginous junction. The remaining 9 cases (81.8%) all branched before the junction (Table 7).

**Table 7:** The number and proportion of cases at different nerve bifurcation locations in type II and variations.

	Type II	Variations	Total	Percentage
Cases	6	5	11	/
At the junction	2	0	2	18.2%
Before the junction	4	5	9	81.8%

Due to the type results having some similarities and differences, the corresponding supplement and modification in the definition of the types are proposed. Firstly, the definition of type II should be changed after emerging from the nerve exit point, the main branch of the nerve has one nerve proximally and then splits into two main branches before or at the intercartilaginous junction of the upper lateral cartilage and the greater alar cartilage. This supplement makes the definition of Type II more comprehensive. Secondly, there are subtypes of the three basic types, with the classification of subtypes mainly according to the position of the nerve bifurcation points and the size of branches. In type I, there is usually one main branch, but when there are multiple tiny branches on the lateral side of the main branch and the branches arise proximal to the intercartilaginous junction, these cases can be considered as a subtype of type I. In type II, the bifurcation point is important to note to distinguish between subtypes of type I and II, type I can be classified if there are multiple tiny branches on the main branch. If there is only one secondary branch on the main branch, it is a subtype of type II. Subtypes of Type III are relatively easy to identify. The nerve type with two branches split at the exit point can be classified, while the occurrence of small branches on the two main branches can be classified as type III subtype. Thirdly, the new classification of Type IV has three main branches, one nerve proximally and then through two bifurcation points splits into three main branches before or at the cartilaginous junction.

On the distance differences, the range of ‘relatively safe operating area’ is smaller in the Caucasian population. Both Han et al. [12] and Tian et al. [13] proposed this concept previously. The range value of this region is approximately the minimum value of the measured distance multiplied by 2. Hence, according to the results obtained from the measured minima in this study, in Caucasians, the relatively safe region is 10.16 mm, and the safety distance should be limited within 5.08 mm from the midline on each side. This is narrower than for Korean and Chinese populations (13 mm and 10.8 mm, respectively).

The reasons for the above differences are mainly considered as follows: race, embalming method, sample size, gender ratio and age differences. As regards race, the anatomical structure of the nasal bone in Caucasians is longer, larger and thinner than Asians [15, 16]. Furthermore, the junction between the upper lateral cartilage and the nasal bone is longer [17]. Moreover, Caucasians generally have a narrower and more projecting nose shape than Asians [2, 18]. Besides, the embalming methods, using 10% formalin or the Thiel method results in different degrees of dehydration to achieve a certain preservative effect compared with fresh tissue. For example, the cartilages of the noses were softer with different degrees of inward depression in this study and the separation of nasal superficial tissue and exposure of the nerve was more difficult as the soft tissue was more adherent to the cartilage. Therefore, the data collection in fresh tissue is likely to be more accurate compared with the samples treated with any preservative measures. However, compared with formalin-embalmed tissue, Thiel embalming has obvious advantages in terms of soft tissue flexibility and color retention [19]. It has superior proximity to living tissue in nervous cutaneous preservation over formalin-embalmed tissue [20]. Due to the different sample sizes, the distance mean value and the distribution range may also be affected. The external nasal nerve is a relatively small and distal nerve, and it is also difficult to avoid damage during the dissection. Therefore, it is an objective problem that the sample size collected in each study is not very large, however, it is also an unavoidable factor.

Even within the same race, male and female noses have obvious differences in morphology [21]. In Caucasian populations, the size of the male nose is wider, larger and higher than the female nose [22]. Moreover, among Caucasian females, the bony base width is the narrowest, not only narrower than the average of Asian races but also narrower than the average of Caucasian races [23]. But in the record from Tian et al. [13], the proportion of males is three times that of females, and this could mean that their results are more inclined to male data than the overall population average. Additionally, during the aging process, human nasal cartilages will change and the nasal bones may become brittle and cause changes in the underlying bone framework [24, 25]. Therefore, since both the lateral nasal cartilages and the nasal bone are changing with age, the exit point of the external nasal nerve may also change. The difference of the average age between this study and the data from Han et al. [12] is more than 10 years, which may also be an influencing factor of the distance value.

In rhinoplasty, some effective intraoperative nerve protection and relative postoperative treatment can be considered. According to the anatomical results of this study, the operation suggests being within 5.08 mm of the nasal midline on each side. When designing the surgical incision, the direction being more distal and parallel to the nasal midline may avoid severing the main branch of the nerve. Besides, the small notch at the edge of the nasal bone is a good anatomical landmark of the exit point of the nerve. When separating the periosteum and the perichondrium at the nasal dorsum, the periosteum is attached to the bone more tightly than the perichondrium to the cartilage, making it more difficult to separate [13]. Therefore, when doing the separation operation at the nasal dorsum, sharp separation can be used in the area near the small notch, and blunt separation can be used in the area far away from the small notch when the notch can be distinguished, so as to avoid the sudden detachment of force during separation cause the pull-apart of the main branch. Furthermore, as the nerve travels adherent to the perichondrium and within the deep fatty layer, local anesthesia can be carried out at the nerve running site. Local anesthesia thickens the connective and loose tissue, and the swollen soft tissue encloses the nerve, which can reduce the damage to the nerve during separation [13]. At the same time, local infiltration anesthesia has a certain hemostatic effect on the operative area, reducing the blurred operative area caused by bleeding. Moreover, the technique of endoscopy assisted rhinoplasty has the advantage of fewer injuries and faster recovery [26]. In addition, if it is inevitable to cut the nerve during the operation, it is also necessary to avoid the main branch or cut off at its distal end as much as possible, or to preserve the integrity of at least one side of the nerve as much as possible. However, in the case of known intraoperative nerve injury, intraoperative platelet-rich fibrin (PRF) coverage or local injection of postoperative platelet-rich plasma (PRP) can be attempted [27–30]. It is also necessary to communicate well with patients before the surgery about the symptoms that may occur after surgery. Besides, the records of nerve morphology presented here may also provide a basis for the external nasal nerve self-repair after injury. Furthermore, the range of distance can also be used as a reference for the nerve block.

## Conclusions

It can be concluded that there are obvious anatomical differences between the external nasal nerves of the Caucasian and Asian populations. These differences are manifested in the nerve types and the distance between the external nasal nerve exit point and the nasal midline. There are three main differences in types: nerve bifurcation points, subtypes and type IV. In terms of distances, the average is larger, and the distribution range of the exit point is wider in Caucasian population. However, the minimum value is smaller which means that the region of ‘relatively safe operating area’ is narrower. Therefore, Caucasians may be more likely to suffer nerve damage during rhinoplasty than Asians.
